# Hsp-27 levels and thrombus burden relate to clinical outcomes in patients with ST-segment elevation myocardial infarction

**DOI:** 10.18632/oncotarget.17852

**Published:** 2017-05-13

**Authors:** Maozhou Tian, Lingmin Zhu, Hongyang Lin, Qiaoyan Lin, Peng Huang, Xiao Yu, Yanyan Jing

**Affiliations:** ^1^ Department of Cardiac Surgery, The Affiliated Yantai Yuhuangding Hospital of Qingdao University, Yantai, Shangdong 264000, P.R. China; ^2^ Department of Cardiology, The Affiliated Yantai Yuhuangding Hospital of Qingdao University, Yantai, Shangdong 264000, P.R. China; ^3^ Emergency Centre, The Affiliated Yantai Yuhuangding Hospital of Qingdao University, Yantai, Shangdong 264000, P.R. China; ^4^ Department of Endocrinology, The Affiliated Yantai Yuhuangding Hospital of Qingdao University, Yantai, Shangdong 264000, P.R. China

**Keywords:** Hsp-27, thrombus burden, STEMI, major adverse cardiovascular event

## Abstract

High thrombus burden, subsequent distal embolization, and myocardial no-reflow remain a large obstacle that may negate the benefits of urgent coronary revascularization in patients with ST-segment elevation myocardial infarction (STEMI). However, the biological function and clinical association of Hsp-27 with thrombus burden and clinical outcomes in patients with STEMI is not clear. Consecutive patients (*n* = 146) having STEMI undergoing primary percutaneous coronary intervention (pPCI) within 12 hours from the onset of symptoms were enrolled in this prospective study in the Affiliated Yantai Yuhuangding Hospital of Qingdao University, Yantai, Shangdong, P.R. China. Patients were divided into low thrombus burden and high thrombus burden groups. The present study demonstrated that patients with high-thrombus burden had higher plasma Hsp-27 levels ([32.0 ± 8.6 vs. 58.0 ± 12.3] ng/mL, *P* < 0.001). The median value of Hsp-27 levels in all patients with STEMI was 45 ng/mL. Using the receiver operating characteristic (ROC) curve analysis, plasma Hsp-27 levels were of significant diagnostic value for high thrombus burden (AUC, 0.847; 95% CI, 0.775–0.918; *P* < 0.01). The multivariate cox regression analysis demonstrated that Hsp-27 > 45 ng/mL (HR 2.801, 95% CI 1.296–4.789, *P* = 0.001), were positively correlated with the incidence of major adverse cardiovascular events (MACE). Kaplan-Meier survival analysis demonstrated that MACE-free survival at 180-day follow-up was significantly lower in patients with Hsp-27 > 45 ng/mL (log rank = 10.28, *P* < 0.001).

Our data demonstrate that plasma Hsp-27 was positively correlated with high thrombus burden and the incidence of MACE in patients with STEMI who underwent pPCI.

## INTRODUCTION

Intracoronary thrombus formation due to the rupture of an atherosclerotic plaque and subsequently reducing coronary blood flow constitute the main pathophysiology underlying ST-segment elevation myocardial infarction (STEMI) [1]. Primary percutaneous coronary intervention (pPCI) of the infarct-related artery in patients with STEMI should be referred for thrombolytic therapy as soon as possible [1]. However, high thrombus burden may increase infarct size by blocking microvascular areas and causing deterioration of ventricular function during PCI [2]. The high thrombus burden is an independent predictor of major adverse cardiovascular event (MACE) and infarct-related artery stent thrombosis (IRA-ST) in patients treated with drug-eluting stent (DES) for STEMI [3]. However, the mechanisms of plaque thrombus remain unclear and the clinical importance and impact have been underestimated due to limited resolution of intravascular ultrasound (IVUS) and computed tomography [4]. Identifying predictors of the intracoronary thrombus burden may contribute to improved management of intracoronary thrombi in patients with STEMI undergoing pPCI.

Heat shock proteins (HSPs), which are abundant intracellular proteins found in both prokaryotic and eukaryotic organisms, act as chaperones and are involved in protein folding and transport [5]. A considerable amount of experimental evidence indicates that increasing expression of HSPs leads to an important cardio-protective role in ischemia/reperfusion injury, heart failure, cardiac hypertrophy, and arrhythmia, implicating these molecules as potential innovative therapeutic agents [6]. Heat shock protein 27 (Hsp-27) is abundant in cardiac and skeletal muscles and increases in response to stress in order to protect against insults such as ischemia [7–9] Hsp-27 is a 27-kDa chaperone, which can interact with a large number of different proteins and is induced in response to stress [10]. Plasma Hsp-27 concentrations are elevated in the early hours following ACS, but decrease to levels near to those in healthy individuals after about 12 hours from the onset of chest pain [11]. It is unclear whether Hsp-27 levels are associated with thrombus burden and clinical outcomes in patients with STEMI. The aim of this study is to evaluate the association of Hsp-27 levels with thrombus burden and clinical outcomes in patients with STEMI who underwent pPCI.

## RESULTS

**Figure 1:**
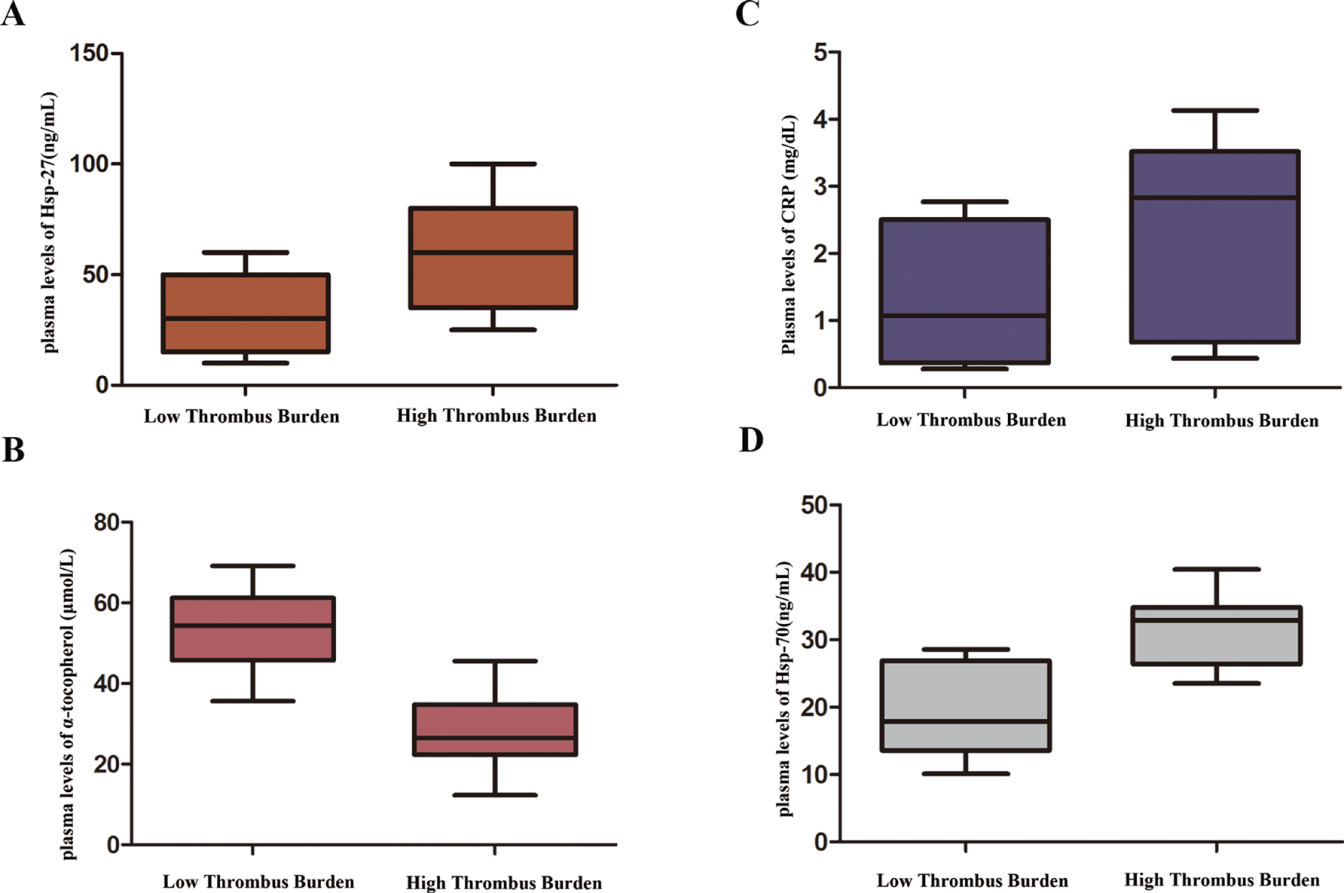
Comparison of plasma heat shock protein 27 (Hsp-27) levels between low and high thrombus burden groups (*P* < 0.01)

**Figure 2:**
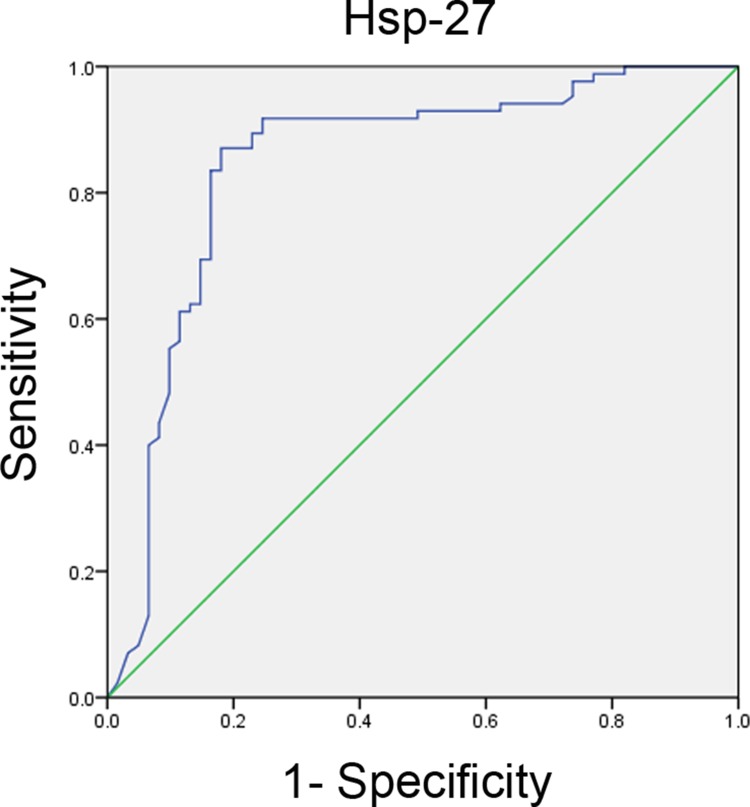
The receiver–operating characteristic (ROC) curve of plasma Hsp-27 for predicting high thrombus burden (AUC, 0.847; 95% CI, 0.775–0.918; *P* < 0.01)

The study population consisted of 146 patients with STEMI. The study cohort consisted of 74 males (50.68%) and 72 females (49.32%), and the mean age of the participants was 62.8 ± 10.5 years. The baseline clinical characteristics between thrombus burden groups are shown in Table 1. Gender, BMI, age, dyslipidemia, DM, smoking, and hypertension were not significantly different between the groups (*P* = 0.441, *P* = 0.501, *P* = 0.617, *P* = 0.558, *P* = 0.122, *P* = 0.120, and *P* = 0.421, respectively). D-Dimer, CK-MB, hemoglobin, platelet count, TC, TG, LDL-C, HDL-C, and white blood cell count were not significantly different between the groups (*P* = 0.650, *P* = 0.145, *P* = 0.478, *P* = 0.437, *P* = 0.325, *P* = 0.368, *P* = 0.386, *P* = 0.486, and *P* = 0.235, respectively). Previous medications, including β-blockers, angiotensin converting enzyme inhibitors (ACEI), angiotensin receptor blockers (ARBs), aspirin, nitrates, statins were not significantly different between the groups (*P* = 0.468, *P* = 0.374, *P* = 0.565, *P* = 0.196, *P* = 0.298, and *P* = 0.380, respectively). Culprit vessels were not significantly different between the groups (left anterior descending, circumflex, and right coronary artery; *P* = 0.768, *P* = 0.283, and *P* = 0.392, respectively). The low thrombus burden and high thrombus burden groups did not differ significantly in the Pain-to-ballon time (308 ± 135 *vs*. 316 ± 112 min, *P* > 0.05) and Door-to-balloon time (80 ± 15 *vs*. 85 ± 20 min, *P* > 0.05) observed in STEMI patients undergoing pPCI. There were no significant differences in the rate of patients treated with stent in low thrombus burden group, compared to that in high thrombus burden group (62 [91.2%] *vs.* 74 [94.9%], *P* > 0.05) as is shown in Table 1. However, patients with low thrombus burden demonstrated significantly higher rates of TIMI grade 3 flow pre-PCI (25 [36.8%] *vs.* 11 [14.1%], *P* < 0.05). Post-PCI coronary flow was slower in patients with pre-PCI high thrombus burden, as demonstrated by lower rates of TIMI grade 3 flow (64 [94.1%] *vs.* 65 [83.3%], *P* < 0.05). As shown in Figure 1A, patients with high-thrombus burden had higher plasma Hsp-27 levels ([32.0 ± 8.6 *vs*. 58.0 ± 12.3] ng/mL, *P* < 0.001). The plasma levels of C-reactive protein (CRP) in the high thrombus burden group were significantly higher than levels in the low thrombus burden group ([1.312 ± 0.319 *vs.* 2.425 ± 0.440] mg/dL, *P* < 0.05; Figure 1B). The plasma α-tocopherol concentrations in the high thrombus burden group were significantly lower than those in the low thrombus burden group ([53.55 ± 6.18 *vs.* 28.04 ± 3.27] μmol/L, *P* < 0.05; Figure 1C). The levels of plasma Hsp-70 were significantly higher in patients with high thrombus burden group than in the low thrombus burden group ([19.16 ± 3.20 *vs.* 31.60 ± 4.52] ng/mL, *P* < 0.05; Figure 1D). Moreover, When statistical analysis was performed in the whole group of combined low thrombus burden and high thrombus burden groups, the plasma levels of Hsp-27 were significantly correlated with the plasma levels of CRP (*r =* 0.454, *P <* 0.05), plasma levels of α-tocopherol (*r =* 0.386, *P <* 0.05) and plasma levels of Hsp70 (*r =* 0.632, *P <* 0.05). Using the receiver operating characteristic (ROC) curve analysis, plasma Hsp-27 levels were of significant diagnostic value for high thrombus burden (AUC, 0.847; 95% CI, 0.775–0.918; *P* < 0.01; Figure 2).

**Table 1:** Baseline clinical characteristics according to thrombus burden^a^

Variable	Low Thrombus Burden (*n* = 68)	High Thrombus Burden (*n* = 78)	*P* value
Male	36 (52.9%)	38 (48.7%)	0.441
BMI (kg/m^2^)	23 ± 2.2	24 ± 2.5	0.501
Age (years)	62.5 ± 11.3	63.2 ± 10.2	0.617
Hypertension	24 (35.3%)	30 (38.5%)	0.421
DM	18 (26.5%)	19 (24.4%)	0.122
Dyslipidemia	30 (44.1%)	35 (44.9%)	0.558
Smoking	30 (44.1%)	36 (46.2%)	0.120
TC (mg/dL)	189 ± 39	172 ± 65	0.325
TG (mg/dL)	109 ± 25	102 ± 18	0.368
LDL-C (mg/dL)	118 ± 32	106± 22	0.386
HDL-C (mg/dL)	45 ± 7.2	50 ± 6.8	0.486
White blood cell count, × 10^9^ /L	10.0 ± 4.2	11.2 ± 3.0	0.235
Platelet count, × 10^9^ /L	223 ± 56	218 ± 46	0.437
Hemoglobin, g/dL	14.2 ± 2.6	14.4 ± 2.8	0.478
D-Dimer (μg/L)	128.72 ± 98.58	112.26 ± 86.53	0.650
CK-MB (IU/L)	156 ± 103	171 ± 126	0.145
Medications			
β-blockers	12 (17.6%)	13 (16.7%)	0.468
ACEI	19 (27.9%)	21 (26.9%)	0.374
ARB	19 (27.9%)	24 (30.7%)	0.565
Aspirin	9 (13.2%)	12 (15.4%)	0.196
Nitrates	16 (23.5%)	18 (23.1%)	0.298
Statins	27 (39.7%)	36 (46.2%)	0.380
Culprit vessel			
LAD	34 (50.0%)	42 (53.8%)	0.768
RCA	22 (32.4%)	26 (33.3%)	0.283
LCX	12 (17.6%)	10 (12.8%)	0.392
patients treated with stent	62 (91.2%)	74 (94.9%)	0.156
Door-to-ballon time (min)	80 ± 15	85 ± 20	0.369
Pain-to-ballon time (min)	308 ± 135	316 ± 112	0.278
TIMI grade 3 flow pre-PCI	25 (36.8%)	11 (14.1%)	< 0.05
TIMI grade 3 flow post- PCI	64 (94.1%)	65 (83.3%)	< 0.05

^**a**^Values are given as means ± SD or absolute numbers with relative frequencies.

Abbreviations: BMI, body mass index; DM, Diabetes mellitus; TC, total cholesterol; TG, triglyceride; LDL-C, low-density lipoprotein cholesterol; HDL-C, high-density lipoprotein cholesterol; CK-MB, creatine kinase-MB; ACEI, angiotensin converting enzyme inhibitors; ARB, angiotensin receptor blockers; CCB, calcium channel blockers; LAD, left anterior descending artery; LCX, left circumflex artery; RCA, right coronary artery.

The median value of Hsp-27 levels in all patients with STEMI was 45 ng/mL. Univariate and multivariate logistic regression analyses were performed to determine the independent predictors of Hsp-27 > 45 ng/mL (Table 2). The variables univariately associated with Hsp-27 > 45 ng/mL were D-Dimer (OR 0.865, 95% CI 0.648–0.963, *P* = 0.045), CK-MB (OR 0.756, 95% CI 0.401–0.809, *P* = 0.036), smoking (OR 1.645, 95% CI 1.148–4.128, *P <* 0.001), WBC (OR 1.135, 95% CI 1.035–1.428, *P =* 0.040), high thrombus burden (OR 2.156, 95% CI 1.636–5.082, *P <* 0.001), CRP (OR 0.612, 95% CI 0.358–0.926, *P =* 0.043), cTnI (OR 3.528, 95% CI 1.286–8.059, *P <* 0.001), aspirin (OR 0.879, 95% CI 0.571–0.954, *P =* 0.039). The multivariate logistic regression analysis demonstrated that high thrombus burden (OR 3.260, 95% CI 1.268–6.362, *P <* 0.001), smoking (OR 1.678, 95% CI 1.012–4.796, *P =* 0.020), and cTnI (OR 2.829, 95% CI 1.025–6.426, *P =* 0.002) were positively correlated with incidence of Hsp-27 > 45 ng/mL. Patients with high thrombus burden had a much higher risk of Hsp-27 > 45 ng/mL than patients with low thrombus burden (Table 2).

**Table 2:** Effects of variables on the Hsp-27 > 45 ng/mL in univariate and multivariate logistic regression analyses

Variables	OR (95% CI)	*P* Value
Univariate analysis		
D-Dimer	0.865 (0.648–0.963)	0.045
CK-MB	0.756 (0.401–0.809)	0.036
Smoking	1.645 (1.148–4.128)	*<* 0.001
WBC	1.135 (1.035–1.428)	0.040
High thrombus burden	2.156 (1.636–5.082)	*<* 0.001
CRP	0.612 (0.358–0.926)	0.043
cTnI	3.528 (1.286–8.059)	*<* 0.001
Aspirin	0.879 (0.571–0.954)	0.039
Multivariate analysis		
High thrombus burden	3.260 (1.268–6.362)	0.001
Smoking	1.678 (1.012–4.796)	0.020
cTnI	2.829 (1.025–6.426)	0.002

Abbreviations: CI, confidence interval; OR, odds ratio; WBC, White blood cell; CK-MB, creatine kinase MB; CRP, C-reactive protein; cTnI, Cardiac troponin T.

**Figure 3:**
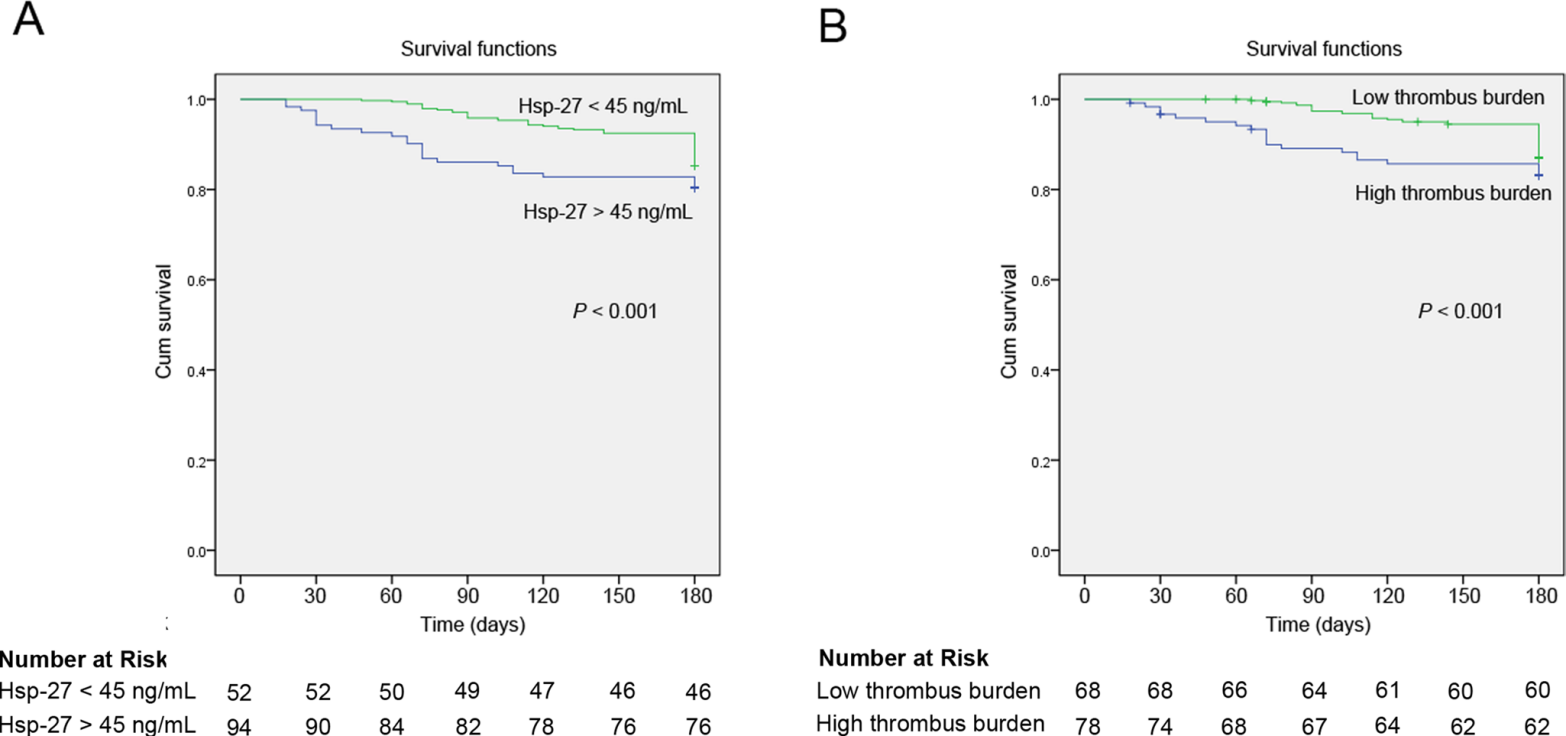
(**A**) Kaplan-Meier major adverse cardiovascular event (MACE)-free survival curves at 180-day follow-up for patients between Hsp-27 > 45 ng/mL and Hsp-27 < 45 ng/mL (log rank = 10.28, *P* < 0.001). (**B**) Kaplan-Meier major adverse cardiovascular event (MACE)-free survival curves at 180-day follow-up for patients between high thrombus burden and low thrombus burden (log rank = 8.36, *P* < 0.001).

**Figure 4:**
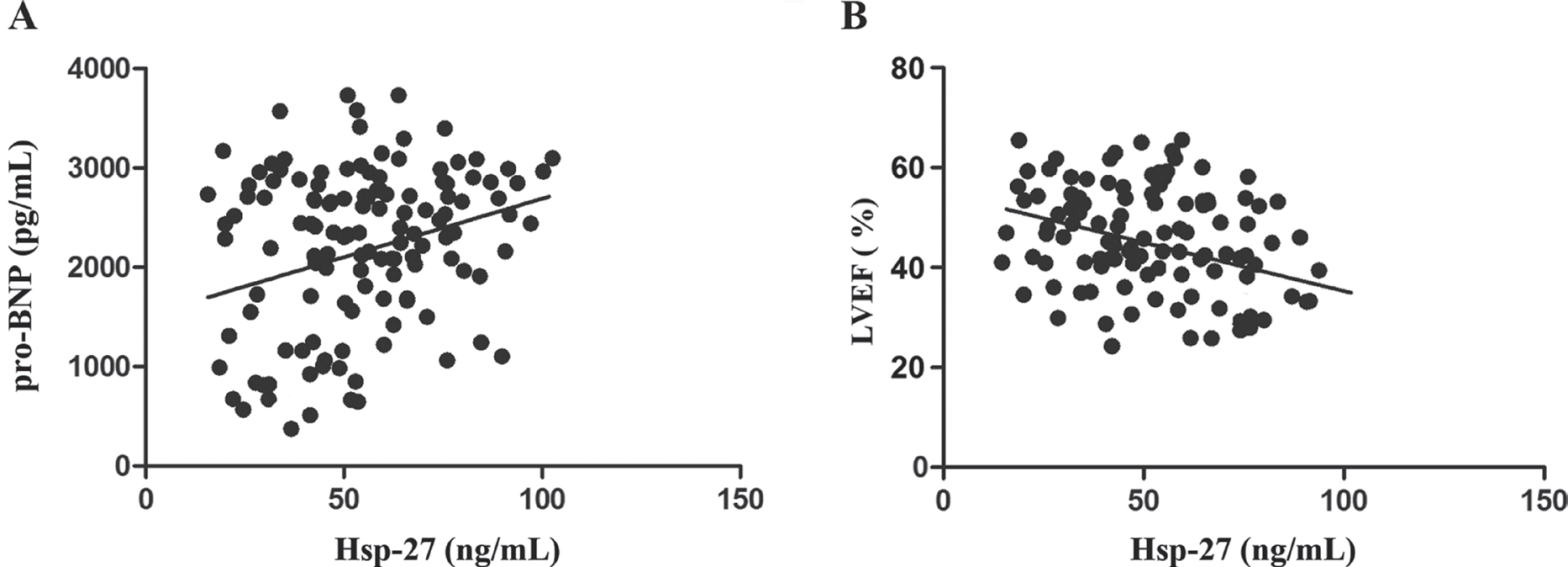
Patterns of circulating plasma Hsp-27 levels in STEMI patients (**A**) Correlations between plasma Hsp-27 levels and pro-BNP in STEMI patients (*r* = 0.624, *P* < 0.05). (**B**) Correlations between plasma Hsp-27 levels and left ventricular ejection fraction (LVEF) in STEMI patients (*r* = −0.528, *P* < 0.05). STEMI, ST-segment elevation myocardial infarction.

At 180-day follow-up, the high thrombus burden group demonstrated a significant trend toward increased incidence of death (4 [5.9%] *vs*. 7 [9.0%], *P* < 0.05). Moreover, differences were noted between the Hsp-27 < 45 ng/mL and Hsp-27 > 45 ng/mL group in death (3 [5.8%] *vs.* 8 [8.5%], *P* < 0.05). Univariate and multivariate cox regression analyses were performed to determine the independent predictors of MACE at 180-day follow-up for patients (Table 3). The univariately associated variables with MACE were Hsp-27 > 45 ng/mL (HR 2.945, 95% CI 1.839–6.451, *P <* 0.001), dyslipidemia (HR 1.780, 95% CI 1.307–2.280, *P =* 0.046), hypertension (HR 1.759, 95% CI 1.314–2.029, *P =* 0.025), DM (HR 1.212, 95% CI 1.012–1.968, *P =* 0.045), cTnI (HR 1.232, 95% CI 1.105–1.809, *P =* 0.048), smoking (HR 1.748, 95% CI 1.340–3.148, *P <* 0.001), high thrombus burden (HR 2.665, 95% CI 1.540–5.276, *P <* 0.001), aspirin (HR 0.709, 95% CI 0.326–0.854, *P =* 0.026), statins (HR 0.469, 95% CI 0.203–0.814, *P =* 0.021), ARB (HR 0.356, 95% CI 0.108–0.614, *P =* 0.018) (Table 3). The multivariate cox regression analysis demonstrated that Hsp-27 > 45 ng/mL (HR 2.801, 95% CI 1.296–4.789, *P =* 0.001), high thrombus burden (HR 2.620, 95% CI 1.240–4.542, *P =* 0.001), smoking (HR 1.672, 95% CI 1.471–2.994, *P =* 0.032) were positively correlated with incidence of MACE. Patients with Hsp-27 > 45 ng/mL had a much higher risk of MACE than patients with Hsp-27 < 45 ng/mL (Table 3). Kaplan-Meier survival analysis demonstrated that MACE-free survival at 180-day follow-up was significantly lower in patients with Hsp-27 > 45 ng/mL (log rank = 10.28, *P* < 0.001; Figure 3A) or with high thrombus burden (log rank = 8.36, *P* < 0.001; Figure 3B). Correlation analysis showed that Hsp-27 levels was positively correlated with pro-BNP (*r* = 0.624, *P* < 0.05; Figure 4A) and negatively correlated with LVEF from echocardiography (*r* = −0.528, *P* < 0.05; Figure 4B).

**Table 3:** Univariate and multivariate cox regression analyses of major adverse cardiovascular events

Variables	HR (95% CI)	*P* Value
Univariate analysis		
Hsp-27 > 45 ng/mL	2.945 (1.839–6.451)	*<* 0.001
Dyslipidemia	1.780 (1.307–2.280)	0.046
Hypertension	1.759 (1.314–2.029)	0.025
DM	1.212 (1.012–1.968)	0.045
CTnI	1.232 (1.105–1.809)	0.048
Smoking	1.748 (1.340–3.148)	*<* 0.001
High thrombus burden	2.665 (1.540–5.276)	*<* 0.001
Aspirin	0.709 (0.326–0.854)	0.026
Statins	0.469 (0.203–0.814)	0.021
ARB	0.356 (0.108–0.614)	0.018
Multivariate analysis		
Hsp-27 > 45 ng/mL	2.801 (1.296–4.789)	0.001
High thrombus burden	2.620 (1.240–4.542)	0.001
Smoking	1.672 (1.471–2.994)	0.032

Abbreviations: DM, Diabetes mellitus; cTnI, Cardiac troponin T; ARB, angiotensin receptor blockers.

## DISCUSSION

Hsp-27 constituted essential components of protein synthesis system and function as molecular chaperones to prevent proteins from misfolding and aggregating [12]. Moreover, it is now well established that Hsp-27 play important cardioprotective roles in myocardial ischemia reperfusion pathological settings [12]. The response of the post-infarcted heart to ischemia in an experimental model of ischemia and reperfusion injury early after acute myocardial infarction (AMI) was significantly associated with 1.7 fold increase in the expression of the Hsp-27 in AMI hearts vs SHAM [13]. The present results showed that plasma Hsp-27 levels and high thrombus burden were independent predictors of clinical outcomes in patients with STEMI who underwent pPCI. Recognition of molecular chaperone and cardioprotective functions of Hsp-27 is fostering active investigations into the potential of Hsp-27 as therapeutic targets for STEMI undergoing primary PCI. Gaining insight into recent advances may have profound implications in the development of Hsp-27-based clinical studies on AMI patients.

High thrombus burden, subsequent distal embolization, and myocardial no-reflow remain a large obstacle that may negate the benefits of urgent coronary revascularization in patients with STEMI. Higher thrombus grade is associated with larger infarct size and slightly worse LV function [14]. High thrombus burden was an independent predictor of mortality (hazard ratio [HR] 1.76, *P* = 0.023) and MACE (HR 1.88, *P* = 0.001). Reduced high thrombus burden by glycoprotein IIb/IIIa antagonists or thrombectomy improved in both epicardial and microvascular perfusion, and was associated with improved ST-segment resolution by 90 minutes [15]. Intracoronary thrombus management remains an important issue to be resolved. Therefore, in this study, we investigated the relationship between Hsp-27 levels and thrombus burden, and whether as a potential novel biomarker for prognosis in patients with STEMI. The present study demonstrated that Hsp-27 > 45 ng/mL and high thrombus burden, were positively correlated with incidence of MACE by multivariate cox regression analysis. MACE-free survival at 180-day follow-up was significantly lower in patients with Hsp-27 > 45 ng/mL or with high thrombus burden analyzed by Kaplan-Meier survival analysis.

Hsp-27, which belongs to the family of small HSPs that bind denatured proteins following cell stress to prevent aggregation, is an abundant protein selectively expressed in myocardial muscle cells. Hsp-27 renders itself an excellent candidate for cardioprotection against apoptosis, infarction, and oxidative stress during myocardial ischemic injury or hypoxic stress [16]. Previous studies suggested that overexpression of Hsp-27 prevents apoptosis by ischemia-reoxygenation in myocardial cells and that overexpression of Hsp-27 in transgenic mice protected the heart against the damaging effects of ischemia/reperfusion [17]. Hsp-27 expression may protect the heart against post-ischemic contractile dysfunction via stabilizing myofilaments, in particular cTnI and cTnT [17]. Additionally, investigators observed that patients with dilated cardiomyopathy (DCM) exhibit a two-fold increase in Hsp-27 expression in the heart compared to healthy control patients [18].

MI usually occurs when the fibrous cap overlying an atherosclerotic plaque in a coronary artery ruptures. The resulting exposure of blood to the atherosclerotic material then triggers thrombus formation, which occludes the artery. Plaque accumulation can impair blood flow, causing health complications, escalating to the grave consequence of plaque “rupture,” exposing pro-thrombotic material from the plaque to the blood and causing occlusion of the artery [19]. However, the biological function and clinical association of Hsp-27 with thrombus burden in patients with STEMI is not clear. Several lines of evidence support the emerging role of Hsp-27 in modulating atheroprogression, by altering the inflammatory components of atherosclerotic plaques and subsequent thrombus formation [20, 21]. Cardiopulmonary bypass had no significant effect on the induction of changes in anti-Hsp-27 levels,which may be due to the formation of immune complexes of antigen–antibody, and antibody levels were higher at the time of discharge [22]. However, serum levels of Hsp-27 are increased in patients undergoing on-pump CABG operation as compared with off-pump CABG technique, owing to spillage of known immune modulatory and apoptosis-associated proteins after CABG operation [23]. Patients with acute cardiac chest pain on admission and approximately 12 h after the acute event had significantly higher Hsp-27 antibody levels than controls [24]. The present study demonstrated that patients with high-thrombus burden had higher plasma Hsp-27 levels (Figure 1A), and high thrombus burden was an independent predictor of Hsp-27 levels > 45 ng/mL (Table 2). The plasma Hsp-27 levels were of significant diagnostic value for high thrombus burden by the ROC curve analyses (Figure 2).

Hsp-27 belongs to a family of widespread stress proteins termed small Hsps (sHsps), which is found particularly in several cell types such as cardiomyocytes, endothelial cells, leukocytes and platelets involving in cardiovascular disease [25]. In addition to its chaperoning functions, Hsp-27 also appears to be involved in a diverse range of cellular functions, promoting cell survival through effects on the apoptotic pathway and plays important roles in myocardial infarction and atherosclerosis [25]. Hsp-27 is highly expressed in the heart, and it has been previously studied in myocardial protection models [26, 27]. Hsp-27 which has been shown to localize on the sarcomere in cardiomyocytes, comes into circulation from destroyed cardiac cells in the infarct zone of the myocardium during early acute myocardial infarction [28]. Martin *et al* reported that decreasing the level of endogenous Hsp-27 resulted in an enhancement of the damaging effects of a subsequent ischemic stimulus [29, 30]. Ghayour-Mobarhan *et al.* have previously reported that Hsp-27 antibody titers were found to be raised in the first 12 h after the onset of acute myocardial infarction, but decreased to normal in the second 12 h, which was similar to our results in the present study [31]. Plasma Hsp-27 antibody titres are high in patients with chest pain who have suffered an acute myocardial infarction or have unstable angina, being higher in the former, and possibly related to the extent of myocardial ischemia or necrosis [31]. It was also reported that an increased expression of Hsp-27 from infarcted myocardial tissue was released into the peripheral circulation several hours following a stroke [25]. Moreover, Hsp-27, which is also expressed by endothelial cells and may stimulate autoimmune response, has roles in stabilizing atherosclerotic plaque, inhibition of plaque development and inhibition of endothelial cell injury through different mechanisms [32, 33]. Additionally, Hsp-27, also present in platelets, may be more involved in controlling actin polymerization during the platelet shape change and subsequent aggregation, which involving in thrombus formation in STEMI [34]. Hsp-27 may also play roles in inhibition of platelet aggregation, cardio-protection in ischemic events and enhancing post ischemic outcome [34, 35].

There is close causal link between high levels of Hsp-27 in patients’ plasma and high risks of thrombus burden. Firstly, Plasma Hsp-27 levels are high in patients with STEMI who have suffered the high thrombus burden or low thrombus burden, being higher in the former, and possibly related to the extent of myocardial ischemia or necrosis [31]. Hsp-27 was found to be raised partly due to the high thrombus burden, which caused the more heavy myocardial ischemia or necrosis compared the low thrombus burden group, but not as ‘thrombogenic’ protein. The higher plasma Hsp-27 levels and high thrombus burden during early STEMI were positively correlated with incidence of MACE at 180-day follow-up in the present study. Secondly, The fact that the higher expression of Hsp-27 levels was observed in the high thrombus burden group than low thrombus burden is probably because this is where the inflammatory processes and oxidative stress were more active [31]. Compared to the high thrombus burden group, the decrease of Hsp-27 expression in the low thrombus burden may be due to an increase of proteolytic activity in region of the plaque. The proteolytic activity has also been described to play an important role in the thrombotic occlusion of the coronary artery on the grounds of atherosclerotic plaque, which is considered the ultimate step in STEMI [36]. Ventura *et al.* demonstrated that the lower levels of Hsp-27 expressed by atherosclerotic plaques could be a result of its degradation by proteolysis [21]. There is a significant relationship between Hsp-27 concentrations in plasma and the proteolytic activity [21]. The increase of Hsp-27 expression in high thrombus may be due to a decrease of proteolytic activity in this region of heavy thrombus [37]. Patients with high-thrombus burden had higher plasma Hsp-27 levels ([32.0 ± 8.6 *vs*. 58.0 ± 12.3] ng/mL, *P* < 0.001) in the present study. Finally, the increase of Hsp-27 releasing from leukocytes and platelets to blood was also related to the complexity of the thrombus formation on atherosclerotic plaques in high thrombus burden [36].

It is interesting and possible to artificially decrease the risk of thrombus burden by targeting the Hsp-27 in patients with STEMI. Firstly, statins (atorvastatin) could decrease the Hsp-27 levels of the proteins secreted by cultured atherosclerotic plaques as well as serum cholesterol [38]. Plasma Hsp-27 concentrations have also been reported to be correlated with total serum cholesterol concentrations in patients with acute coronary syndrome [20]. Interestingly, Hsp-27 detected by PD-Quest analysis as differentially released by atherosclerotic plaques significantly decreased after addition of atorvastatin compared with that in atheroma plaque supernatants in the absence of atorvastatin, in agreement with the proteomic analysis [38]. Statins are also capable of decreasing the levels of different inflammatory proteins in both carotid atherosclerotic plaques and in the blood of patients with carotid atherosclerosis [39]. Secondly, SHIN et al cardiac rehabilitation therapy (CRT) and a combination of CRT with stain treatment (COM) reduced antibody titers to Hsp-60 and Hsp-70 in patients with coronary artery disease (CAD) after percutaneous coronary intervention [40]. There was a significant correlation between antibody titers to Hsp-27 versus Hsp-60 and Hsp-70, involving in autoimmune process of atherosclerosis [40]. Considering the fact that antibody titers to Hsp-27 are associated with the autoimmune process in CAD, CRT and COM may exert greater effects on reduction in plasma Hsp-27 levels in patients with STEMI undergoing PCI than statin treatment only [40]. Based on the findings obtained in the present study and accumulating evidences, down-regulation of the antibody titers to Hsp-27 by cardiac rehabilitation therapy and statins, intervention consisting of cardiac rehabilitation and statin therapy in STEMI patients was found to be of benefit [40]. Finally, use of abciximab was possible to decrease the risk of thrombus burden by targeting the Hsp-27 releasing from platelets of coronary thrombus in patients with STEMI. Abciximab, a platelet glycoprotein (GP) IIb/IIIa inhibitor, in combination with administration of thrombolytics has been shown to reduce angiographically evident thrombus in patients with STEMI [15]. Hsp-27 has important roles in platelet function [34]. There is evidence suggesting a potential role for Hsp-27 in the regulation of transglutaminase activity in stabilizing fibrin–platelet clots. Phosphorylation of Hsp-27 may also contribute to the inhibitory effects of cGK on platelet function [35]. Hsp-27 may be involved in platelet aggregation by modulating actin polymerization [35].

Our study has several limitations, including the small number of patients in a single center in China. Secondly, we could not explain the exact mechanisms triggering change in Hsp-27 between low thrombus burden and high thrombus burden in patients with STEMI undergoing pPCI. Lastly, although we assessed the plasma Hsp-27 levels before PCI, serial measurements of Hsp-27 might be more useful for evaluating changes in inflammatory status, estimating risk during the follow-up period. Therefore, a multicenter trial with a large study population is necessary to validate the clinical importance of Hsp-27 in the future.

In conclusion, the present study results showed that plasma Hsp-27 was positively correlated with high thrombus burden and the incidence of MACE in patients with STEMI who underwent pPCI. Measuring plasma Hsp-27 levels may substantially improve the risk stratification of high thrombus burden and clinical outcomes in STEMI patients.

## MATERIALS AND METHODS

### Study population

Consecutive patients (*n* = 146) having STEMI within 12 hours from onset of symptoms were prospectively enrolled in the study in the Affiliated Yantai Yuhuangding Hospital of Qingdao University, Yantai, Shangdong, P.R. China from June 2014 to January 2016. PCI was performed in all patients and transradial coronary intervention was the preferred operating approach. STEMI was defined as typical chest pain > 30 minutes duration with ST-segment elevation > 0.1 mV in at least 2 consecutive leads on the electrocardiogram or new-onset left bundle branch block [41]. Important exclusion criteria included previous myocardial infarction (MI), previous use of thrombolytic agents for index MI, cardiogenic shock, cardiomyopathy, previous stroke within the past 6 months, and known bleeding diathesis [41]. Clinical data, including baseline clinical characteristics, angiographic, and laboratory features were recorded for all patients. All patients gave their informed consent, and the study protocol was approved by the Yantai Yuhuangding Hospital’s ethics committee.

### Laboratory analysis

Venous blood samples were collected before PCI in all patients. Whole blood was immediately collected into a tube containing ethylene diaminetetraacetate (EDTA); Then, it was centrifuged at 3000 × g for 15 min at 4°C. After separation, plasma was frozen at −80°C until detection for Hsp-27 levels.. Laboratory assessments included total cholesterol (TC), triglycerides (TG), low-density lipoprotein cholesterol (LDL-C), high-density lipoprotein cholesterol (HDL-C), D-Dimer, creatine kinase-MB (CK-MB), and cardiac troponin T (cTnT) levels in the emergency department. The CK-MB assessments were repeated every 6 hours to determine peak levels of CK-MB, unless clinical events suggested further measurements until discharge. Hemoglobin, white blood cells, mean platelet volume, and platelet counts were measured using a Coulter LH 780 Hematology Analyzer (Beckman Coulter Inc, Miami, FL, USA). proBNP plasma levels were measured in each patient on admission by UniCel DXI800 Access Immunoassay System (Beckman Coulter). Plasma Hsp-27 and Hsp-70 levels were determined using sandwich enzyme-linked immunosorbent assay (ELISA) kits (R&D Systems, Minneapolis, MN, USA) and a microplate spectrophotometer (Multiskan Spectrum; Thermo Scientific, Waltham, MA, USA) [42]. All ELISA tests were performed according to the manufacturer’s instructions. Samples were measured in duplicates. The plasma α-tocopherol concentration was measured by high performance liquid chromatography (HPLC) with the use of a reverse-phase column (NovaPak C18, 8100-mm Radial-Pak Cartridge, Waters) [43]. C-reactive protein (CRP) was measured by ELISA according to the manufacturer’s instructions (R&D Systems, Minneapolis, MN, USA). Researchers performing the assays and data analyses were blinded to the groups associated with each sample.

### PCI procedure

All patients also received loading with aspirin (300 mg) and clopidogrel (300 mg) in the emergency department. Left ventricular ejection fraction (LVEF) was measured by 2-dimensional echocardiogram with Simpson methods before primary PCI. A 100 U/kg intravenous bolus of unfractionated heparin was administered through the arterial access sheaths to all patients before the procedure, and anticoagulation level was adjusted to maintain an activated clotting time of 250 to 300 seconds. Coronary angiography and intervention started within 30 minutes after admission using radial artery approach. Coronary arteries were visualized in left and right oblique planes with cranial and caudal angles at 30 frames/s. Thrombus burden was assessed as previously defined by the thrombolysis in myocardial infarction (TIMI) study group, and quantitative angiography was performed in an angiographic core laboratory by investigators blinded to treatment assignment [15]. In TIMI thrombus grade 0, no cineangiography characteristics of thrombus are present; in TIMI thrombus grade 1, possible thrombus is present, with angiography characteristics such as reduced contrast density, haziness, irregular lesion contour, or a smooth convex “meniscus” at the site of total occlusion suggestive; in TIMI thrombus grade 2, there is definite thrombus, with greatest dimensions ≤ 1/2 the vessel diameter; in TIMI thrombus grade 3, there is definite thrombus but with greatest linear dimension .> 1/2 but, < 2 vessel diameters; in TIMI thrombus grade 4, there is definite thrombus, with the largest dimension ≥ 2 vessel diameters; and in TIMI thrombus grade 5, there is total occlusion [15]. The patients were divided into 2 groups with low and high thrombus burden, respectively. The patients were considered to high thrombus burden if TIMI thrombus grades 4 to 5 were present, and the patients with TIMI thrombus grades 1 to 3 were defined as the lower thrombus burden group [44]. In the present study, the study population undergoing primary PCI was stented with drug-eluting stents (DES), Aspirin and clopidogrel were maintained at 100 and 75 mg/d, respectively. Clopidogrel (75 mg/d) was given for 12 months after DES implantation according to the current guideline [3].

### Clinical definitions and follow-up

The diagnosis of diabetes mellitus (DM) was based on previous history of DM treated with or without drugs. Hypercholesterolemia was diagnosed by total cholesterol concentration ≥ 200 mg/dL, and hypertension was defined as systolic pressure ≥ 140 mm Hg and/or a diastolic pressure ≥ 90 mm Hg at least 2 times or if the individual was taking antihypertensive medication [45]. Patients were defined smokers if they smoked ≥ 1 cigarette/d at the time of admission or in the preceding 12 months [45]. Follow-up was performed through the outpatient department and telephone interviews at 180 days after discharge at the discretion of the attending physician. The primary endpoints were combined MACE (including death, reinfarction, and target vessel revascularization [TVR]), LVEF, life-threatening arrhythmia (including ventricular fibrillation or sustained ventricular tachycardia), and heart failure at 180-day follow-up. All patients have reached 180-day follow-up, the time period during which dual anti-platelet therapy was recommended in all patients.

### Statistical analysis

Statistical analysis was performed using SPSS 20.0 software (IBM, USA). Continuous data are presented as mean ± SD. Categorical variables are presented as counts and percentages. Normally distributed data were analyzed using Student *t* test and for non-normally distributed data nonparametric Mann-Whitney test was used. Categorical variables were compared by chi-square test. Correlations between variables were determined by Pearson tests. Univariate and multivariate logistic regression analyses were used to determine independent predictors of Hsp-27 > 45 ng/mL. Receiver operating characteristic (ROC) curve analysis and comparison of the derived area under the curve (AUC) were performed to assess the Hsp-27 as a predictor for high thrombus burden. Univariate and multivariate cox regression analyses were performed to determine independent predictors of MACE. Values were expressed as odds ratio (OR) or hazard ratio (HR), with 95% confidence interval (CI) and *P* values. Kaplan-Meier survival was calculated to evaluate the MACE-free survival at 180-day follow-up, with the difference analyzed by the log-rank test. Statistical significance was set at *P* < 0.05.
